# Prescribing cannabis for harm reduction

**DOI:** 10.1186/1477-7517-9-1

**Published:** 2012-01-01

**Authors:** Mark Collen

**Affiliations:** 1 PainExhibit.com, 9008 El Cajon Way, #4, Sacramento, CA, 95826, USA

**Keywords:** cannabis, cannabinoids, opioids, neuropathic pain, chronic pain, harm reduction, ethics

## Abstract

Neuropathic pain affects between 5% and 10% of the US population and can be refractory to treatment. Opioids may be recommended as a second-line pharmacotherapy but have risks including overdose and death. Cannabis has been shown to be effective for treating nerve pain without the risk of fatal poisoning. The author suggests that physicians who treat neuropathic pain with opioids should evaluate their patients for a trial of cannabis and prescribe it when appropriate prior to using opioids. This harm reduction strategy may reduce the morbidity and mortality rates associated with prescription pain medications.

## 

Neuropathic pain (NP) is defined as pain caused by a lesion or disease of the central or peripheral somatosensory nervous system [1]. NP affects between 5% and 10% of the US population [2] and examples include diabetic neuropathy, complex regional pain syndrome, radiculopathy, phantom limb pain, HIV sensory neuropathy, multiple sclerosis-related pain, and poststroke pain [3]. Neuropathic pain is difficult to treat and opioid analgesics are often prescribed [4]. Recent science has demonstrated efficacy in treating NP with cannabis, [5-7] a safer drug than opioids [8]. This paper suggests that physicians who treat neuropathic pain should prescribe cannabis prior to using opioids as a harm reduction (HR) strategy. Topics covered include how harm reduction applies to prescription opioid substitution, the legality of medicinal cannabis, a comparison of cannabis to opioids, the science on treating NP with cannabis and cannabinoids, and the ethics of prescribing a drug which is deemed illegal on the federal but not the state level.

Medicine relies upon the principle of, "First, do no harm," and one might supplement the axiom to read - "First, do no harm, and second, reduce all the harm you can." "Harm reduction" or "harm minimization" can be defined in the broadest sense as strategies designed to reduce risk or harm [9]. Those harmed may include the individual, others impacted by the harmed person, and society [9]. The substitution of a safer drug for one that is more dangerous is considered harm reduction [10]. Specific examples of HR include prescribing methadone or buprenorphine to replace heroin, [11] prescribing nicotine patches to be used instead of smoking tobacco, [12] and prescribing intranasal naloxone to patients on opioid therapy to be utilized in case of overdose [13]. Substituting cannabis for prescribed opioids may be considered a harm reduction strategy.

Under the Federal Controlled Substance Act "marihuana" is illegal and classified as a schedule I substance-meaning it has a high potential for abuse and no accepted medical use [14]. However, sixteen states and the District of Columbia have legalized cannabis for medicinal use and these include Alaska, Arizona, California, Colorado, Delaware, Hawaii, Maine, Michigan, Montana, Nevada, New Jersey, New Mexico, Oregon, Rhode Island, Vermont, and Washington [15]. Each state law differs but all allow physicians to "authorize" or "recommend" cannabis for specific ailments [16]. This "recommendation" affords legal protections for patients to obtain and use medicinal cannabis, and may be considered the "prescription."

Cannabis (C*annabis sativa*) and the opium poppy (*Papaver somniferum*) are both ancient plants that have been used medicinally for thousands of years [17,18]. The natural and synthetic derivatives of opium, including morphine, are called "opioids." [19] "Cannabinoids" is the term for a class of compounds within cannabis of which delta-9-tetrahydrocannabinol (THC) is the most familiar [20]. Besides THC, approximately 100 other cannabinoids have been identified [21,22] including one of special scientific interest called "cannabidiol" (CBD) [23]. The human body produces both endogenous cannabinoids (endocannabinoids) and opioids (endorphins) and contains specific receptors for these substances [24,25]. There is an extensive literature on opioids but far less on cannabis/cannabinoids (CC).

Adverse effects from opioids include respiratory depression, sedation, sleep disturbance, cognitive and psychomotor impairment, delirium, hallucinations, seizures, hyperalgesia, constipation, nausea, and vomiting [26-28]. Adverse events from cannabis/cannabinoids include psychotic episodes, anxiety or panic reactions, memory impairment, reduced concentration, disorientation, lowered blood pressure and increased heart rate [7,29-31]. In a systematic review Wang and colleagues found most adverse events for short-term cannabis use were not serious, and there was a lack of evidence to determine adverse effects for long-term use [32]. Opioids and cannabis share issues of addiction, physical dependence, tolerance and withdrawal [5,33,34].

Between 1999 and 2006 approximately 65,000 people died from opioid analgesic overdose [35]. Regarding fatal overdose from cannabis, Carter and colleagues write, "... this well documented fact: no one has ever died from an overdose of cannabis." [8] In addition, there is insufficient data to demonstrate smoking cannabis causes lung cancer [36] but long-term use is associated with an increased risk of respiratory problems [37]. Although, eating cannabis [38] avoids the respiratory issues. In 2001 the total cost of prescription opioid abuse was estimated at $8.6 billion [39]. Unfortunately, there are no comprehensive studies on the total cost of cannabis abuse. However, enforcing the prohibition on cannabis costs an estimated $7.7 billion per year [40]. Since the federal and most state governments view any use of cannabis as abuse - including medicinal use - one might include this cost. According to a report from the Substance Abuse and Mental Health Services Administration between 1999 and 2009 admissions for treatment of nonheroin opioid abuse increased approximately 516% while admissions for cannabis saw a 53% rise [41].

Nerve pain can be refractory to treatment [42] and opioids are often used as a second-line therapy while antidepressants and anticonvulsants are commonly used first [4,43]. Moreover, opioids may provide only limited pain relief and as Henry McQuay writes, "...you may be able to decrease neuropathic pain with strong opioids, but the decrease is often slight and is achieved with an adverse eﬀect burden that will not be tolerable over weeks to months." [44] Cannabis and cannabinoid research is in its relative infancy and many studies are of short duration and with small sample sizes [6]. However, a number of review articles suggest that treating neuropathic pain with cannabis/cannabinoids is efficacious and with moderate adverse effects [5-7]. The most thorough of the systematic reviews was of randomized controlled trials (RCTs) of CC therapy [6] which looked at nine studies [45-53] whose focus was on treating different types of neuropathic pain with either smoked cannabis, [45-48] a synthetic cannabinoid similar to THC, [49,50] or a whole plant extract of THC and CBD in a 1:1 ratio [51-53]. CBD may moderate the psychoactive effect of THC and have analgesic properties [24]. Seven of the nine studies demonstrated efficacy for using CC for neuropathic pain [45-49,52,53] while two had mixed results, [50,51] and eight of the nine studies found no serious adverse events [45-51,53].

A closer look at the four RCTs which evaluated smoked cannabis for neuropathic pain [45-48] reveals some common and contrasting elements (Table 1). Two of the studies, Ware et al. [45] and Wilsey et al., [47] examined cannabis in treating a variety of NP conditions; while the other two, Ellis et al. [46] and Abrams et al., [48] explored the effects of cannabis on HIV-related neuropathic pain. Both Wilsey et al. [47] and Abrams et al. [48] required participants to have previously used cannabis in order to reduce the risk of adverse reactions from psychoactive effects. The RCTs used cannabis with a variety of THC strengths ranging from 0% for placebo [45-48] to 9.4% in Ware et al. [45] Each study required participants to continue taking their regular medications during the cannabis trials and all found a significant decrease in pain compared to placebo [45-48]. In addition, adverse events were tolerable for the vast majority of participants [45-48].

**Table 1:** Comparison of Randomized Controlled Trials of Smoked Cannabis for Neuropathic Pain

	**Ware et al**. [45]	**Wilsey et al**. [47]	**Ellis et al**. [46]	**Abrams et al**. [48]
**Purpose of Study**	"... explore the safety and efficacy of smoked cannabis in outpatients with chronic neuropathic pain."	"... examine whether smoking cannabis produces dose-dependent analgesia on both spontaneous and evoked pain in patients with neuropathic pain."	"... ascertain a safe, clinically useful, and efficacious dosing range for smoked medicinal cannabis as a short-term analgesic in the treatment of refractory neuropathic pain in HIV DSPN."	"... determine the effect of smoked cannabis on the neuropathic pain of HIV-SN, and to determine if cannabinoids have a more general analgesic and anti-hyperalgesic effect."
**Study Design**	randomized, double-blind, placebo-controlled, crossover	randomized, double-blind, placebo-controlled, crossover	randomized, double-blind, placebo-controlled, crossover	randomized, double-blind, placebo-controlled
**Sample Size**	21	38	28	50
**Pain Conditions**	NP caused by trauma or surgery, with allodynia or hyperalgesia	spinal cord injury, peripheral NP, and multiple-sclerosis	HIV infection, refractory NP	HIV infection, HIV-SN
**Exclusion Criteria**	active substance abuse, pain due to cancer or nociceptive causes, history of psychosis, cardiac or pulmonary disease, pregnant	active substance abuse, major depressive disorder, schizophrenia, bipolar disorder, cardiovascular or pulmonary disease, never having used cannabis	active substance abuse, history of psychosis or intolerance to cannabinoids, serious medical conditions	active substance abuse, serious medical conditions, never having used cannabis
**Cannabis Potency (%THC)**	2.5%, 6%, 9.4%; 0% placebo	3.5%, 7%; 0% placebo	ranging from 1% to 8%; 0% placebo	3.56%; 0% placebo
**Length of Study**	4 phases over 8 weeks	3 sessions over ~ 4 weeks	5 study phases over 7 weeks	4 phases over 3 weeks
**Findings**	The 9.4% THC dose significantly decreased pain as compared to placebo. Patients also experienced improved sleep, decreased depression and anxiety as compared to placebo.	Both the 3.5% and 7% THC cannabis produced equal and significant analgesia compared to placebo. Pain was more tolerable at higher doses compared to placebo. Both doses had no effect on evoked pain or allodynia.	Pain reduction was significantly greater compared to placebo. Mood, physical disability, quality of life and sleep improved during all study treatments. The majority of patients titrated to the highest THC dose of 8%.	Patients experienced a significant decrease in pain compared to placebo. Cannabis decreased induced hyperalgesia but had little impact on heat stimulation.
**Adverse Events**	AE increased with potency. The most frequent complaints from the highest dose cannabis was headache, dry eyes, burning sensation, dizziness numbness and cough.	Both doses produced a general cognitive decline with the highest dose causing the greatest impairment. Both groups felt "high" or "stoned."	2 patients dropped out due to AE; 1 had a psychotic episode, 1 had respiratory irritation. AE was greater for cannabis than placebo and included cognitive impairment, fatigue, sedation, sleepiness, dry mouth, thirst.	2 patients had AE (1 dizziness; 1 anxiety) which were treated with a one-time dose of lorazepam. AE included anxiety, disorientation, confusion and dizziness.

HIV-DSPN, HIV distal sensory polyneuropathy; HIV-SN, HIV-associated sensory neuropathy; NP, neuropathic pain; AE, adverse events

Commentators have suggested that patients should use whole plant cannabis, as opposed to chemical derivatives, because of other potentially beneficial compounds [8,19]. In addition, a number of articles have reported on interactions between cannabinoid and opioid receptors which may result in enhanced analgesia and a synergistic effect when CC is added to opioids [54,55]. This may translate into patients being able to reduce their opioid intake with adjuvant cannabinoid therapy [5,29].

Although prescribing cannabis is legal in 16 states and the District of Columbia, it remains illegal at the federal level. Portions of the American Medical Association's Code of Medical Ethics, Opinion 1.02 - The Relation of Law and Ethics reads, "Ethical values and legal principles are usually closely related, but ethical obligations typically exceed legal duties. In some cases, the law mandates unethical conduct." "In exceptional circumstances of unjust laws, ethical responsibilities should supersede legal obligations." [56] An "exceptional circumstance of unjust laws" may be interpreted as the federal ban on cannabis for medical use. Sixteen states and the District of Columbia found the federal government's prohibition on prescribing and using medicinal cannabis so unjust as to create laws in direct violation of federal statute. Therefore, one could surmise that prescribing cannabis for the purpose of harm reduction is ethical even though it violates federal law. In addition, Hayry suggests that the idea of "freedom" also provides an ethical reason for prescribing cannabis and he writes, "... whatever the legal situation, respect for the freedom of the individual would imply that requests like this (for medicinal cannabis) should be granted, either by health professionals, or by society as a whole." [57]

In states where medicinal cannabis is legal, physicians who treat neuropathic pain with opioids should evaluate their patients for a trial of cannabis and prescribe it when appropriate prior to using opioids. There is sufficient evidence of safety and efficacy for the use of CC in the treatment of nerve pain relative to opioids and as Carter et al write, "From a pharmacological prospective, cannabinoids are considerably safer than opioids..." [8] Prescribing cannabis in place of opioids for neuropathic pain may reduce the morbidity and mortality rates associated with prescription pain medications and may be an effective harm reduction strategy.

[The subject of cannabis dosing is beyond the scope of this paper but those interested should consider reading Carter GT, Weydt P, Kyashna-Tocha M, Abrams DI. **Medicinal cannabis: rational guidelines for dosing**. *IDrugs *2004;**7**:464-70.]

## References

[B1] International Association for the Study of Pain IASP Taxonomy http://www.iasp-pain.org/AM/Template.cfm?Section=Pain_Defi...isplay.cfm&ContentID=1728#Neuropathicpain, Accessed December 2, 2011

[B2] Center for Medicinal Cannabis Research, University of California Report to the Legislature and Governor of the State of California presenting findings pursuant to SB847 which created the CMCR and provided state funding 2010

[B3] Dworkin RH Backonja M Rowbotham MC Allen RR Argoff CR Bennett GJ Bushnell MC Farrar JT Galer BS Haythornthwaite JA Hewitt DJ Loeser JD Max MB Saltarelli M Schmader KE Stein C Thompson D Turk DC Wallace MS Watkins LR Weinstein SM Advances in neuropathic pain: diagnosis, mechanisms, and treatment recommendations Arch Neurol 2003 60 1524 34 10.1001/archneur.60.11.1524 14623723

[B4] Dworkin RH O'Connor AB Audette J Baron R Gourlay GK Haanpää ML Kent JL Krane EJ Lebel AA Levy RM Mackey SC Mayer J Miaskowski C Raja SN Rice AS Schmader KE Stacey B Stanos S Treede RD Turk DC Walco GA Wells CD Recommendations for the pharmacological management of neuropathic pain: an overview and literature update Mayo Clin Proc 2010 85 Suppl 3 S3 14 20194146 10.4065/mcp.2009.0649 PMC2844007

[B5] Leung L Cannabis and its derivatives: review of medical use J Am Board Fam Med 2011 24 452 62 10.3122/jabfm.2011.04.100280 21737770

[B6] Lynch ME Campbell F Cannabinoids for Treatment of Chronic Non-Cancer Pain; a Systematic Review of Randomized Trials Br J Clin Pharmacol 2011 in press 10.1111/j.1365-2125.2011.03970.x PMC3243008 21426373

[B7] Thaler A Gupta A Cohen SP Cannabinoids for pain management Adv Psychosom Med 2011 30 125 38 21508629 10.1159/000324070

[B8] Carter GT Flanagan AM Earleywine M Abrams DI Aggarwal SK Grinspoon L Cannabis in palliative medicine: improving care and reducing opioid-related morbidity Am J Hosp Palliat Care 2011 28 297 303 10.1177/1049909111402318 21444324

[B9] Kleinig J The ethics of harm reduction Subst Use Misuse 2008 43 1 16 10.1080/10826080701690680 18189202

[B10] Reiman A Cannabis as a substitute for alcohol and other drugs Harm Reduct J 2009 6 35 10.1186/1477-7517-6-35 19958538 PMC2795734

[B11] Sacerdote P Franchi S Gerra G Leccese V Panerai AE Somaini L Buprenorphine and methadone maintenance treatment of heroin addicts preserves immune function Brain Behav Immun 2008 22 606 13 10.1016/j.bbi.2007.12.013 18294814

[B12] Bailey SR Fong DM Bryson SW Fortmann SP Killen JD Perceived drug assignment and treatment outcome in smokers given nicotine patch therapy J Subst Abuse Treat 2010 39 150 6 10.1016/j.jsat.2010.05.013 20598833 PMC2910821

[B13] Wermeling DP Opioid harm reduction strategies: focus on expanded access to intranasal naloxone Pharmacotherapy 2010 30 627 31 10.1592/phco.30.7.627 20575626

[B14] United States Drug Enforcement Administration Lists of: Scheduling Actions, Controlled Substances, Regulated Chemicals http://www.deadiversion.usdoj.gov/schedules/orangebook/orangebook.pdf, Accessed December 2, 2011

[B15] The National Organization for the Reform of Marijuana Laws Active State Medical Marijuana Programs http://norml.org/index.cfm?Group_ID=3391, Accessed August 21, 2011

[B16] Medical Marijuana. ProCon.org 16 Legal Medical Marijuana States and DC Laws, Fees, and Possession Limits http://medicalmarijuana.procon.org/view.resource.php?resourceID=000881#details, Accessed December 2, 2011

[B17] Russo EB History of cannabis and its preparations in saga, science, and sobriquet Chem Biodivers 2007 4 1614 48 10.1002/cbdv.200790144 17712811

[B18] Brownstein MJ A brief history of opiates, opioid peptides, and opioid receptors Proc Natl Acad Sci USA 1993 90 5391 3 10.1073/pnas.90.12.5391 8390660 PMC46725

[B19] Duarte DF Opium and opioids: a brief history Rev Bras Anestesiol 2005 55 135 46 19471817

[B20] Di Marzo V A brief history of cannabinoid and endocannabinoid pharmacology as inspired by the work of British scientists Trends Pharmacol Sci 2006 27 134 40 10.1016/j.tips.2006.01.010 16476494

[B21] Mehmedic Z Chandra S Slade D Denham H Foster S Patel AS Ross SA Khan IA ElSohly MA Potency trends of Δ9-THC and other cannabinoids in confiscated cannabis preparations from 1993 to 2008 J Forensic Sci 2010 55 1209 17 10.1111/j.1556-4029.2010.01441.x 20487147

[B22] Russo EB Taming THC: potential cannabis synergy and phytocannabinoid-terpenoid entourage effects Br J Pharmacol 2011 163 1344 64 10.1111/j.1476-5381.2011.01238.x 21749363 PMC3165946

[B23] Russo E Guy GW A tale of two cannabinoids: The therapeutic rationale for combining tetrahydrocannabinol and cannabidiol Med Hypotheses 2006 66 234 46 10.1016/j.mehy.2005.08.026 16209908

[B24] Placzek EA Okamoto Y Ueda N Barker EL Mechanisms for recycling and biosynthesis of endogenous cannabinoids anandamide and 2-arachidonylglycerol J Neurochem 2008 107 987 1000 18778304 10.1111/j.1471-4159.2008.05659.x PMC2581634

[B25] Bodnar RJ Endogenous opiates and behavior: 2009 Peptides 2010 31 2325 59 10.1016/j.peptides.2010.09.016 20875476

[B26] Vella-Brincat J Macleod AD Adverse effects of opioids on the central nervous systems of palliative care patients J Pain Palliat Care Pharmacother 2007 21 15 25 10.1080/J354v21n01_05 17430825

[B27] Furlan AD Sandoval JA Mailis-Gagnon A Tunks E Opioids for chronic noncancer pain: a meta-analysis of effectiveness and side effects CMAJ 2006 174 1589 94 10.1503/cmaj.051528 16717269 PMC1459894

[B28] Bushlin I Rozenfeld R Devi LA Cannabinoid-opioid interactions during neuropathic pain and analgesia Curr Opin Pharmacol 2010 10 80 6 10.1016/j.coph.2009.09.009 19857996 PMC2818338

[B29] Hosking RD Zajicek JP Therapeutic potential of cannabis in pain medicine Br J Anaesth 2008 101 59 68 10.1093/bja/aen119 18515270

[B30] National Institute on Drug Abuse InfoFacts: Marijuana http://drugabuse.gov/infofacts/marijuana.html, Accessed December 2, 2011

[B31] Schubart CD Sommer IE van Gastel WA Goetgebuer RL Kahn RS Boks MP Cannabis with high cannabidiol content is associated with fewer psychotic experiences Schizophr Res 2011 130 216 21 10.1016/j.schres.2011.04.017 21592732

[B32] Wang T Collet JP Shapiro S Ware MA Adverse effects of medical cannabinoids: a systematic review CMAJ 2008 178 1669 78 10.1503/cmaj.071178 18559804 PMC2413308

[B33] Walwyn WM Miotto KA Evans CJ Opioid pharmaceuticals and addiction: the issues, and research directions seeking solutions Drug Alcohol Depend 2010 108 156 65 10.1016/j.drugalcdep.2010.01.001 20188495 PMC3072810

[B34] Lichtman AH Martin BR Cannabinoid tolerance and dependence Handb Exp Pharmacol 2005 168 691 717 10.1007/3-540-26573-2_24 16596793

[B35] Warner M Chen LH Makuc DM Increase in fatal poisonings involving opioid analgesics in the United States, 1999-2006 NCHS Data Brief 2009 22 1 8 19796521

[B36] Hashibe M Morgenstern H Cui Y Tashkin DP Zhang ZF Cozen W Mack TM Greenland S Marijuana use and the risk of lung and upper aerodigestive tract cancers: results of a population-based case-control study Cancer Epidemiol Biomarkers Prev 2006 15 1829 34 10.1158/1055-9965.EPI-06-0330 17035389

[B37] Tetrault JM Crothers K Moore BA Mehra R Concato J Fiellin DA Effects of marijuana smoking on pulmonary function and respiratory complications: a systematic review Arch Intern Med 2007 167 221 8 10.1001/archinte.167.3.221 17296876 PMC2720277

[B38] Peat S Using cannabinoids in pain and palliative care Int J Palliat Nurs 2010 16 481 5 20972379 10.12968/ijpn.2010.16.10.79211

[B39] Birnbaum HG White AG Reynolds JL Greenberg PE Zhang M Vallow S Schein JR Katz NP Estimated costs of prescription opioid analgesic abuse in the United States in 2001: a societal perspective Clin J Pain 2006 22 667 76 10.1097/01.ajp.0000210915.80417.cf 16988561

[B40] Egan D Miron JA Earleywine M The budgetary implications of marihuana prohibition Pot Politics: Marijuana and the Costs of Prohibition 2007 New York: Oxford University Press 17 39

[B41] Substance Abuse and Mental Health Services Administration Treatment Episode Data Set (TEDS). 1999-2009 2011 Rockville, MD; Substance Abuse and Mental Health Services Administration National Admissions to Substance Abuse Treatment Services, DASIS Series: S-56, HHS Publication No. (SMA) 11-4646

[B42] Rahn EJ Hohmann AG Cannabinoids as pharmacotherapies for neuropathic pain: from the bench to the bedside Neurotherapeutics 2009 6 713 37 10.1016/j.nurt.2009.08.002 19789075 PMC2755639

[B43] Finnerup NB Sindrup SH Jensen TS Recent advances in pharmacological treatment of neuropathic pain F1000 Med Rep 2010 2 52 21170362 10.3410/M2-52 PMC2996861

[B44] McQuay H Evidence-based medicine: what is the evidence that it has made a difference? Palliat Med 2011 25 5 394 7 10.1177/0269216310394707 21708846

[B45] Ware MA Wang T Shapiro S Robinson A Ducruet T Huynh T Gamsa A Bennett GJ Collet JP Smoked cannabis for chronic neuropathic pain: a randomized controlled trial CMAJ 2010 182 E694 701 10.1503/cmaj.091414 20805210 PMC2950205

[B46] Ellis RJ Toperoff W Vaida F van den Brande G Gonzales J Gouaux B Bentley H Atkinson JH Smoked medicinal cannabis for neuropathic pain in HIV: a randomized, crossover clinical trial Neuropsychopharmacology 2009 34 672 80 10.1038/npp.2008.120 18688212 PMC3066045

[B47] Wilsey B Marcotte T Tsodikov A Millman J Bentley H Gouaux B Fishman S A randomized, placebo-controlled, crossover trial of cannabis cigarettes in neuropathic pain J Pain 2008 9 506 21 10.1016/j.jpain.2007.12.010 18403272 PMC4968043

[B48] Abrams DI Jay CA Shade SB Vizoso H Reda H Press S Kelly ME Rowbotham MC Petersen KL Cannabis in painful HIV-associated sensory neuropathy: a randomized placebo-controlled trial Neurology 2007 68 515 21 10.1212/01.wnl.0000253187.66183.9c 17296917

[B49] Karst M Salim K Burstein S Conrad I Hoy L Schneider U Analgesic effect of the synthetic cannabinoid CT-3 on chronic neuropathic pain: a randomized controlled trial JAMA 2003 290 1757 62 10.1001/jama.290.13.1757 14519710

[B50] Frank B Serpell MG Hughes J Matthews JN Kapur D Comparison of analgesic effects and patient tolerability of nabilone and dihydrocodeine for chronic neuropathic pain: randomised, crossover, double blind study BMJ 2008 336 199 201 10.1136/bmj.39429.619653.80 18182416 PMC2213874

[B51] Berman JS Symonds C Birch R Efficacy of two cannabis based medicinal extracts for relief of central neuropathic pain from brachial plexus avulsion: results of a randomised controlled trial Pain 2004 112 299 306 10.1016/j.pain.2004.09.013 15561385

[B52] Wade DT Robson P House H Makela P Aram J A preliminary controlled study to determine whether whole-plant cannabis extracts can improve intractable neurogenic symptoms Clin Rehabil 2003 17 21 9 10.1191/0269215503cr581oa 12617376

[B53] Nurmikko TJ Serpell MG Hoggart B Toomey PJ Morlion BJ Haines D Sativex successfully treats neuropathic pain characterised by allodynia: a randomised, double-blind, placebo-controlled clinical trial Pain 2007 133 210 20 10.1016/j.pain.2007.08.028 17997224

[B54] Welch SP Interaction of the cannabinoid and opioid systems in the modulation of nociception Int Rev Psychiatry 2009 21 143 51 10.1080/09540260902782794 19367508

[B55] Desroches J Beaulieu P Opioids and cannabinoids interactions: involvement in pain management Curr Drug Targets 2010 11 462 73 10.2174/138945010790980303 20017728

[B56] American Medical Association Code of Medical Ethics. Opinion 1.02-The Relation of Law and Ethics http://www.ama-assn.org/ama/pub/physician-resources/medical-ethics/code-medical-ethics/opinion102.page?, Accessed December 2, 2011

[B57] Hayry M Prescribing cannabis: freedom, autonomy, and values J Med Ethics 2004 30 333 6 10.1136/jme.2002.001347 15289511 PMC1733898

